# Predicting hemoglobin levels in whole blood donors using transition models and mixed effects models

**DOI:** 10.1186/1471-2288-13-62

**Published:** 2013-05-02

**Authors:** Kazem Nasserinejad, Wim de Kort, Mireille Baart, Arnošt Komárek, Joost van Rosmalen, Emmanuel Lesaffre

**Affiliations:** 1 Department of Biostatistics, Erasmus MC, Rotterdam, the Netherlands; 2 Sanquin Blood Supply, Nijmegen, the Netherlands; 3 Faculty of Mathematics and Physics, Charles University in Prague, Prague, Czech Republic; 4 L-Biostat, KU Leuven, Leuven, Belgium

**Keywords:** Blood donations, Hemoglobin level, Longitudinal data, Panel data, Transition models, Mixed effects models, Prediction, Kalman filter

## Abstract

**Background:**

To optimize the planning of blood donations but also to continue motivating the volunteers it is important to streamline the practical organization of the timing of donations. While donors are asked to return for donation after a suitable period, still a relevant proportion of blood donors is deferred from donation each year due to a too low hemoglobin level. Rejection of donation may demotivate the candidate donor and implies an inefficient planning of the donation process. Hence, it is important to predict the future hemoglobin level to improve the planning of donors’ visits to the blood bank.

**Methods:**

The development of the hemoglobin prediction rule is based on longitudinal (panel) data from blood donations collected by Sanquin (the only blood product collecting and supplying organization in the Netherlands). We explored and contrasted two popular statistical models, i.e. the transition (autoregressive) model and the mixed effects model as plausible models to account for the dependence among subsequent hemoglobin levels within a donor.

**Results:**

The predictors of the future hemoglobin level are age, season, hemoglobin levels at the previous visits, and a binary variable indicating whether a donation was made at the previous visit. Based on cross-validation, the areas under the receiver operating characteristic curve (AUCs) for male donors are 0.83 and 0.81 for the transition model and the mixed effects model, respectively; for female donors we obtained AUC values of 0.73 and 0.72 for the transition model and the mixed effects model, respectively.

**Conclusion:**

We showed that the transition models and the mixed effects models provide a much better prediction compared to a multiple linear regression model. In general, the transition model provides a somewhat better prediction than the mixed effects model, especially at high visit numbers. In addition, the transition model offers a better trade-off between sensitivity and specificity when varying the cut-off values for eligibility in predicted values. Hence transition models make the prediction of hemoglobin level more precise and may lead to less deferral from donation in the future.

## Background

Blood transfusion is an essential part of modern healthcare which helps save millions of lives each year. Since blood is a unique resource for which an artificial substitute has yet to be found, blood donations are in great need. However, occasionally donation cannot be accepted. There may be several reasons for the ineligibility of a blood donor for donation, a common reason being low hemoglobin level of the donor [1,2]. A hemoglobin (Hb) level of 8.4 mmol/l (135 g/l) and 7.8 mmol/l (125 g/l) for men and women, respectively, is widely accepted as the lower cut-off value of eligibility for donation [2-5]. While donors are asked to return for donation after a suitable period, a relevant proportion of blood donors are temporarily deferred from donation each year due to low Hb levels [2]. Rejection of donation may demotivate the candidate donor and implies inefficient planning of the donation process [6,7]. Hence, it is important to predict the future Hb level to improve the planning of donors’ visits to the blood bank. Prediction models for low Hb level deferral have been developed previously [5,8].

The main goal of this paper is to illustrate the use of two well-known longitudinal models in predicting the future Hb level after a visit to the blood bank. An adequate prediction will help the blood bank to apply appropriate interventions (e.g. postponing the next invitation) for blood donation when the Hb value falls below the cut-off value. Prediction is based on models developed using historical data of Hb levels obtained from Sanquin Blood Supply in the Netherlands. More specifically, in this paper we examine the predictive performance of the transition (autoregressive panel data) model and the mixed effects model.

## Methods

### Data

The data have been obtained from Sanquin Blood Supply, which is the only blood product collecting and supplying organization in the Netherlands. In this paper, we analyze newly registered whole blood donors whose first visit to the collection centers occurred in the period between January 1, 2007 and December 31, 2009 and have donated at least twice during this period. Whole blood is a term used in transfusion medicine for a standard blood donation as opposed to plasma and platelet donation. The data were collected from 16,158 newly registered whole blood donors (54.6% women). The reason for selecting this set of blood donors is that they constitute a relatively homogeneous group that did not donate prior to establishing the Sanquin database. We excluded donors who had missing values for the Hb level, and the data of the remaining 15,625 donors were used in the analyses.

**Figure 1:**
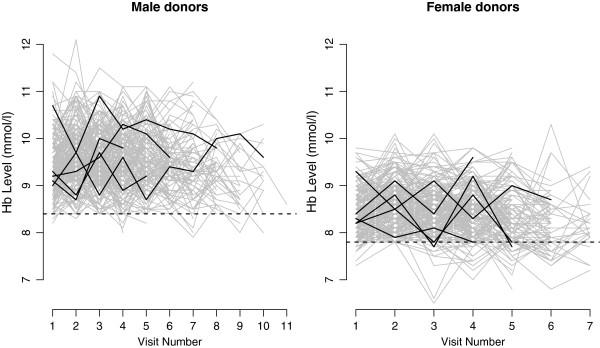
**Hemoglobin levels profile.** Profile of hemoglobin levels for successive visits to the blood bank of a random sample of male and female donors. The profiles of 5 randomly selected donors are highlighted. The dashed horizontal lines show the Hb cut-off values of eligibility for donation.

In Sanquin Blood Supply, a candidate has to register prior to donation; after registration he/she will receive an information package and an invitation to attend a blood donor health check. If the test results are satisfactory, the candidate will be invited to donate blood. Therefore, the first visit to the Sanquin Blood Supply is not a donation but a health check that includes a measurement of the Hb level. After a successful whole blood donation, a male (female) donor is allowed to return for the next donation after a period of at least 8 weeks with a maximum of 5 (3) donations per year. In each visit, prior to donation, the candidates are screened for health risks that might make the donation unsafe for either the donor or the recipient. These tests include taking fingerstick capillary samples for measuring Hb level and filling out a health appraisal form. Based on the results of these tests, the candidate may not be eligible for donation due to a too low Hb level or other reasons that he/she mentioned in the health appraisal form. Finally, eligible candidates will donate 500 milliliters (ml) blood. We defined donation status in each visit as a binary variable in our data set (donation =1, no donation =0). In Figure 1, profiles of the Hb level are displayed for male and female donors separately. The dashed horizontal lines show the corresponding Hb level cut-off points of eligibility for donation.

Several factors are known to be associated with the Hb level and hence may be used as predictors for Hb level, i.e. gender [9], age [9], and body mass index (BMI) [10,11]. In this study, we take into account the effect of gender and age in our models, but we decided to ignore the effect of BMI due to the fact that the BMI was not recorded for approximately 40% of donors. Also, based on a pilot study we found that the impact of BMI on Hb level is secondary. The season in which the visit takes place also affects the Hb level, namely in a warm season Hb level is lower on average [12,13]. Here season is used as a binary covariate, i.e. cold season (= 0) includes fall and winter and warm season (= 1) includes spring and summer. Male and female donors have different Hb profiles, therefore we analyzed the data for men and women separately. Inter-visit intervals differ between donors, in our data set the median inter-visit interval for male donors is 72 (inter-quartile range: 29 − 92) days and for female donors it is 93 (inter-quartile range: 25 − 131) days. In principle, varying intervals between visits require continuous-time models, but these models are beyond the scope of this paper. Therefore we decided to ignore this feature of the data, and we used the sequential number of the visit rather than the actual time of the visit. We also take into account the status of the previous visit (donation or deferral) as a binary covariate in the prediction model. Since no donations have been made prior to the first visit, the value of donation at previous visit (DPV) for the first visit is defined to be ‘no donation’.

This research has been performed with the approval of the ethical advisory council of the Sanquin Blood Supply Foundation. Moreover, all donors have given their consent by stating that part or all of their donations can be used for research aiming at improving the blood supply chain. Our ethical advisory council includes members of both Sanquin and non-Sanquin affiliations. This committee includes members with the background training and experience required for such ethical committees.

### Statistical analysis

Since successive Hb levels on the same subject are correlated, we need to employ statistical models that can take this correlation into account. For this purpose, we applied two well-known models, namely the transition model and the mixed effects model. However, we commence with a multiple linear regression model as a benchmark to show the capability of transition and mixed effects models. These statistical analyses were performed in R version 2.15.2 [14] using the stats package for the multiple linear regression models, the nlme package for the mixed effects models, the KalmanLike and the mle functions in the stats4 package for the transition models, and the mixAK and pROC packages to draw profile and ROC curve plots. We used a significance level of *α* = 0.05 and no correction for multiple testing was implemented.

#### Multiple linear regression model

A naive approach to analyze the successive Hb levels is a multiple linear regression model, in which the current response of a particular subject is regressed only on time-varying covariates, i.e. age, season, and DPV. A multiple linear regression model can be expressed as:

(1) $$y_{\text{it}}=\alpha +\beta_{1}\text{Ag}e_{\text{it}}+\beta_{2}\text{Seaso}n_{\text{it}}+\beta_{3}\text{DP}V_{\text{it}}+\varepsilon_{\text{it}},$$

where *y*_*it*_ is the *t*th observation of the *i*th individual, *α* is an unknown constant (intercept), and the *β*’s are unknown regression coefficients. It is assumed that the residuals *ε*_*it*_ are normally distributed and mutually independent with mean zero and constant variance, i.e., $\varepsilon_{\text{it}}\overset{\text{iid}}{∼}N(0,\sigma_{\epsilon }^{2})$. Due to the fact that this model cannot take into account the intra-subject correlations and the previous Hb levels, it is only presented as a benchmark model to show the capability of transition and mixed effects models.

#### Transition model

A transition model, also known as an autoregressive panel data model in the econometrics literature, is a dynamic regression model, in which the current response of a particular subject (donor) is regressed on previous responses of that subject as well as on other covariates [15]. A transition model of order *q* can be expressed as:

(2) $$\begin{array}{ll} y_{\text{it}}=\alpha & +\beta_{1}\text{Ag}e_{\text{it}}+\beta_{2}\text{Seaso}n_{\text{it}}+\beta_{3}\text{DP}V_{\text{it}}\\ & +\overset{q}{\underset{r=1}{\sum }}\gamma_{r}(y_{\text{it}-r}-(\beta_{1}\text{Ag}e_{\text{it}-r}\\ & \;\;\;\;+\beta_{2}\text{Seaso}n_{\text{it}-r}+\beta_{3}\text{DP}V_{\text{it}-r}))\\ & +\varepsilon_{\text{it}}, \end{array}$$

where *y*_*it*_ is the *t*th observation of the *i*th individual, *α* is an unknown constant, and the *β*’s are unknown regression coefficients, *y*_*it* − *r*_ and (*Age*_*it* − *r*_,*Season*_*it* − *r*_,*DPV*_*it* − *r*_) are *r*th lagged response and covariates, respectively and *γ*_*r*_ is the corresponding coefficient of the *r*th lag. Classically it is assumed that the residuals *ε*_*it*_ are normally distributed and mutually independent with mean zero and constant variance, i.e., $\varepsilon_{\text{it}}\overset{\text{iid}}{∼}N(0,\sigma_{\epsilon }^{2})$. In a transition model with order *q*, the predicted values depend on *q* lagged previous observations; however, to calculate the predicted value using equation 2, there are not enough previous observations for the first few visits of a donor. We employed the method of maximum likelihood via a linear quadratic estimation (Kalman filter) algorithm to estimate the parameters in the transition model. This algorithm enables us to calculate the exact likelihood function, which includes the distribution of the first few observations of each donor [16-18]. As a result, the maximum likelihood estimation also includes the information of donors who have made fewer visits than the order of the transition model.

#### Linear mixed effects model

The linear mixed effects (LME) model which contains a mixture of fixed effects and random effects provides another way to deal with longitudinal responses within a subject. The correlation among responses pertaining to one subject is now induced by introducing random effects, which can be regarded as subject-specific terms [19,20]. A special case of the mixed effects model is the random intercept model which can be expressed as:

(3) $$y_{\text{it}}=\alpha +b_{0i}+\beta_{1}\text{Ag}e_{\text{it}}+\beta_{2}\text{Seaso}n_{\text{it}}+\beta_{3}\text{DP}V_{\text{it}}+\varepsilon_{\text{it}},$$

where *α* is an unknown constant, the *β*’s are regression coefficients (fixed effects) and the *b*_*i*0_ is the random intercept. The random intercept *b*_*i*0_ can be viewed here as the deviation of the *i*th subject-specific mean of Hb levels from the population mean of Hb levels. It is assumed that *b*_0*i*_ and *ε*_*it*_ are normally distributed and mutually independent with mean zero and different constant variances, i.e., $b_{0i}∼N(0,\sigma_{b0}^{2})$, and $\varepsilon_{\text{it}}∼N(0,\sigma_{\epsilon }^{2})$[21]. Furthermore, in the random intercept model the correlation between two observations of a subject is constant and is equal to the intra-class correlation given by $\rho =\frac{\sigma_{b0}^{2}}{\sigma_{b0}^{2}+\sigma_{\epsilon }^{2}}$[19,20].

Although the simplicity of the mixed model with only random intercept is appealing, it poses the restriction that the correlation between the repeated measurements remains constant over time. An extension that allows for a more flexible specification of the covariance structure is a mixed model with random intercept and slope; this model introduces an additional random effects term (e.g. age), and assumes that the rate of change in the covariates (age) differs between subjects. The mixed effects model with random intercept and slope can be expressed as:

(4) $$\;y_{\text{it}}=\alpha +b_{0i}+(b_{1i}+\beta_{1})\text{Ag}e_{\text{it}}+\beta_{2}\text{Seaso}n_{\text{it}}+\beta_{3}\text{DP}V_{\text{it}}+\varepsilon_{\text{it}},$$

where *α* is an unknown constant, the *β*’s contains population-specific parameters. *b*_*i*_ = (*b*_*i*0_,*b*_*i*1_) contains subject-specific parameters (intercept and the effects of age) describing how the evolution of the *i*th individual deviates from the average evolution in the population, and where the residual component $\varepsilon_{i}={\left(\varepsilon_{i1},\ldots ,\varepsilon_{in_{i}}\right)}^{\prime }$ is a vector containing the common error components, with *ε*_**i**_ ∼ *N* (0,*Σ*_*i*_). In this paper, we assumed that $\Sigma_{i}=\sigma^{2}I_{n_{i}}$, so that, conditional on the values of the random effects, a person’s measurements of the Hb level are independent. However, additional correlation among the errors can be accommodated by allowing for a more general covariance structure (e.g., autoregressive) in the model. It is assumed *b*_*i*_ has a bivariate normal distribution with mean zero and a diagonal covariance matrix, so that *ε*_**i**_ and *b*_*i*_ are mutually independent. To estimate the parameters in the mixed effects models we employed the method of restricted maximum likelihood (REML). We applied an empirical Bayes method (EB) to predict a person’s random intercept and slope based on his/her all previous observations [20].

We used a likelihood ratio test to choose between the mixed model with random intercept and the mixed model with random intercept and slope. In this case, the likelihood ratio test statistic for testing a random slope in the model is a mixture of chi-squared distributions with 1 and 2 degrees of freedom [19].

Note that the linear mixed effects model is based on quite different assumptions than the transition model. In principle, if one model is correct, the other model must be wrong. However, in practice we never know the truth and in fact it is possible that both models are wrong. Despite this, we can still check which of the two models performs better in predicting the Hb level.

#### Prediction performance

To avoid a too optimistic assessment of the model predictions by using the data twice, i.e. for model building and parameter estimation as well as model evaluation, we have randomly divided the data set (*n*=15,625 donors) into two parts: a training data set consisting of all observations of 7,709 donors and a validation data set consisting of all observations of the remaining 7,916 donors [22]. The models are estimated using the training data set, and the model predictions are evaluated using the validation data set. We used a dynamic prediction approach in the sense that to predict Hb level at a visit we used the observations of all previous visits, therefore for each visit we updated our prior information. Since no prior information is available for the first visit, the predicted values are based only on the gender and age of the donor and the season in which the visit takes place.

The ultimate purpose of our longitudinal model is to predict future Hb values, given previously measured Hb values of a blood donor. Two criteria for choosing a model are *Akaike’s information criterion* (AIC) [23] and the related *Bayesian information criterion* (BIC) [24]. We report the values of AIC and BIC for the training data set. In addition, we have chosen to estimate the predictive accuracy using some simple and intuitively clear measures, i.e. *mean squared prediction error* (MSPE) as a function of the visit number. At the *t*th visit, the MSPE is computed as:

(5) $$\text{MSP}E_{t}=\overset{N_{t}}{\underset{i=1}{\sum }}\frac{{\left({ŷ}_{\text{it}}-y_{\text{it}}\right)}^{2}}{N_{t}},$$

where ${ŷ}_{\text{it}}$ and *y*_*it*_ are the predicted and observed values, respectively and *N*_*t*_ is the total number of subjects at occasion *t*. *MSPE*_*t*_ is a well-known measure to evaluate prediction. The MSPE values are calculated for the validation data set only.

We also computed the sensitivity and specificity of the predicted values for assessing the eligibility for donation in the validation data set. Specifically, we computed the proportion of individuals that are correctly predicted to be eligible for donation based on the clinical cut-off value (i.e. an Hb level of at least 8.4 mmol/l and 7.8 mmol/l for men and women, respectively). However, one may also optimize the cut-off value for the predicted values to obtain a receiver-operating characteristic (ROC) curve. In this ROC curve, the state variable is a dichotomous variable indicating whether the Hb level is below the clinical cut-off value of 8.4 mmol/l for men or 7.8 mmol/l for women; the test variable is the predicted value ${ŷ}_{\text{it}}$. Varying the cut-off value for the predicted value will change the sensitivity to detect that a donor will be eligible; however the assessment of donors’ eligibility is based on the clinical cut-off value, which is not changed in the ROC analysis. We calculated the area under the curve (AUC) to compare the models. The difference in the AUCs between the models was tested using a bootstrap technique [25,26] that takes into account the correlation between the areas that is induced by the paired nature of the data.

## Results

Table 1 presents descriptive statistics of the training and validation data sets. Different models are applied on the Sanquin data. We start with a multiple linear regression model (*Model LR*) that includes age, season, and donation at previous visit (DPV) as covariates. This model ignores the correlation among the subsequent hemoglobin values and hence is not a candidate choice, however, it serves as a benchmark to evaluate the more realistic models. In addition to the multiple linear regression model, a mixed effects model (*Model LME*) and transition (autoregressive) models of different orders are fitted to the training data set. The transition models are denoted as *Models AR(1) to AR(5)*, where the number indicates the order of the transition model. The data for male donors supported only a mixed model with random intercept (*p*-value =0.19), but the data for female donors supported a mixed model with random intercept and slope (*p*-value <0.001).

**Table 1:** Descriptive statistics of the training and validation data sets

**Data set**	***Gender***	***#Donor***	***#Deferral***	***#Cold Season***	***Age: Mean (SD)***	***Visit: Med (IQR)***
*Training data set*	Male	3610	769 (4.58%)	10213 (50.05%)	34.57 (12.9)	5 (3)
	Female	4306	1596 (9.62%)	10387 (49.71%)	32.66 (12.8)	5 (1)
	**Total**	**7916**	**2365 (7.08)**%	**20600 (49.88%)**	**33.53 (12.9)**	**5 (2)**
*Validation data set*	Male	3449	688 (4.27%)	9781 (49.95%)	34.28 (12.6)	5 (3)
	Female	4260	1729 (10.41%)	10341 (49.54%)	32.77 (12.8)	5 (2)
	**Total**	**7709**	**2417 (7.38%)**	**20122 (49.74%)**	**33.45 (12.7)**	**5 (2)**

Note: SD= Standard deviation, IQR= Interquartile range.

Tables 2 and 3 display the results of the fitted models on the training data set for male and female donors, respectively. These tables indicate that all transition effects (regression coefficients of past Hb values) are significant, although the effect of previous Hb level decreases with the lag. The effect of age is negative for male donors and positive for female donors, these results are consistent with previous studies (e.g. see [5,8]). During warm seasons Hb level is lower on average than during cold seasons; this result is also supported by previous studies (e.g. see [12,13]). Furthermore, our models show that having had a donation in the previous visit has a negative effect on the current Hb level.

**Table 2:** Parameter estimates (standard errors) of the models estimated using the training data set for male donors

**Parameter**	***Model LR***	***AR(1)***	***AR(2)***	***AR(3)***	***AR(4)***	***AR(5)***	***Model LME***
*intercept*	9.6448	9.6309	9.6441	9.6560	9.6617	9.6633	9.6719
	(0.0142)	(0.0206)	(0.0231)	(0.0243)	(0.0246)	(0.0247)	(0.0243)
*Age*	-0.0045	-0.0043	-0.0044	-0.0045	-0.0047	-0.0047	-0.0049
	(0.0003)	(0.0005)	(0.0006)	(0.0006)	(0.0006)	(0.0007)	(0.0006)
*Season*(*Warm*)	-0.0627	-0.0615	-0.0681	-0.0699	-0.0693	-0.0694	-0.0698
	(0.0089)	(0.0074)	(0.0066)	(0.0066)	(0.0067)	(0.0067)	(0.0067)
*DPV*	-0.0610	-0.0469	-0.0350	-0.0385	-0.0440	-0.0474	-0.0636
(*Donation*)	(0.0092)	(0.0089)	(0.0079)	(0.0074)	(0.0072)	(0.0072)	(0.0068)
*γ*_1_	—	0.5158	0.3685	0.3053	0.2746	0.2630	—
	—	(0.0061)	(0.0068)	(0.0076)	(0.0082)	(0.0087)	—
*γ*_2_	—	—	0.2888	0.2080	0.1766	0.1621	—
	—	—	(0.0078)	(0.0087)	(0.0084)	(0.0091)	—
*γ*_3_	—	—	—	0.2207	0.1730	0.1581	—
	—	—	—	(0.0095)	(0.0104)	(0.0109)	—
*γ*_4_	—	—	—	—	0.1488	0.1257	—
	—	—	—	—	(0.0123)	(0.0129)	—
*γ*_5_	—	—	—	—	—	0.0829	—
	—	—	—	—	—	(0.0167)	—

**Table 3:** Parameter estimates (standard errors) of the models estimated using the training data set for female donors

**Parameter**	***Model LR***	***AR(1)***	***AR(2)***	***AR(3)***	***AR(4)***	***AR(5)***	***Model LME***
*intercept*	8.2737	8.2394	8.2555	8.2678	8.2698	8.2702	8.2832
	(0.0123)	(0.0164)	(0.0180)	(0.0186)	(0.0187)	(0.0187)	(0.0181)
*Age*	0.0042	0.0044	0.0042	0.0040	0.0040	0.0040	0.0037
	(0.0003)	(0.0004)	(0.0005)	(0.0005)	(0.0005)	(0.0005)	(0.0005)
*Season*(*Warm*)	-0.0347	-0.0405	-0.0415	-0.0413	-0.0415	-0.0415	-0.0411
	(0.0078)	(0.0062)	(0.0060)	(0.0062)	(0.0061)	(0.0061)	(0.0062)
*DPV*	-0.1106	-0.1411	-0.1273	-0.1307	-0.1335	-0.1346	-0.1387
(*Donation*)	(0.0079)	(0.0075)	(0.0067)	(0.0064)	(0.0063)	(0.0063)	(0.0060)
*γ*_1_	—	0.4669	0.3457	0.3012	0.2878	0.2830	—
	—	(0.0062)	(0.0067)	(0.0074)	(0.0080)	(0.0084)	—
*γ*_2_	—	—	0.2573	0.1963	0.1793	0.1693	—
	—	—	(0.0080)	(0.0088)	(0.0089)	(0.0099)	—
*γ*_3_	—	—	—	0.1742	0.1486	0.1360	—
	—	—	—	(0.0100)	(0.0112)	(0.0121)	—
*γ*_4_	—	—	—	—	0.0831	0.0623	—
	—	—	—	—	(0.0157)	(0.0182)	—
*γ*_5_	—	—	—	—	—	0.0681	—
	—	—	—	—	—	(0.0264)	—

The AIC and BIC values for different models based on the training data set and the MSPE values based on the validation data set are shown in Table 4 for men and women. The results in Table 4 show that, for both genders, AIC and BIC prefer a 5th order transition model over transition models that use fewer lagged observations. However, if we include all models, the smallest AIC and BIC value for the data of female donors are obtained with the mixed model with random intercept and random slope.

**Table 4:** AIC, BIC, and MSEP values for different models for both genders based on the training data set

	**Male donors**			**Female donors**		
**Model**	**AIC**	**BIC**	**MSPE**	**AIC**	**BIC**	**MSPE**
Linear Regression	37087.8	37127.2	4.14	35968.9	36008.6	2.29
Mixed Effects	30524.3	30571.6	2.90	30058.0	30113.6	1.75
AR(1)	32051.0	32098.3	3.07	31559.1	31606.7	1.81
AR(2)	30936.4	30991.6	2.85	30664.7	30720.3	1.73
AR(3)	30471.9	30535.0	2.78	30375.1	30438.7	1.71
AR(4)	30342.5	30413.4	2.78	30341.7	30413.2	1.72
AR(5)	30321.4	30400.2	2.79	30325.1	30404.5	1.72

Note: Lower values of AIC, BIC, and MSEP indicate better model fit.

**Figure 2:**
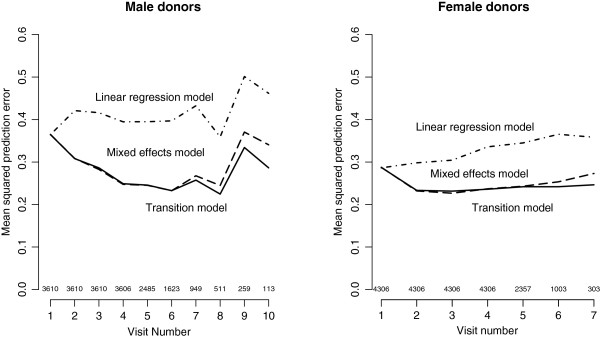
**Mean squared prediction error.** Mean squared prediction error of the linear regression model, the linear mixed effects model, and the 5th order transition model, as a function of the visit number. The included numbers of individuals are displayed above the horizontal axis.

The assessment of predictive accuracy based on MSPE confirms that all transition models and the mixed effects (LME) model provide much better predictions than the multiple linear regression model. In addition, the results indicate that the transition model usually provides a better prediction than the mixed effects model, especially at high visit numbers, see Figure 2.

**Figure 3:**
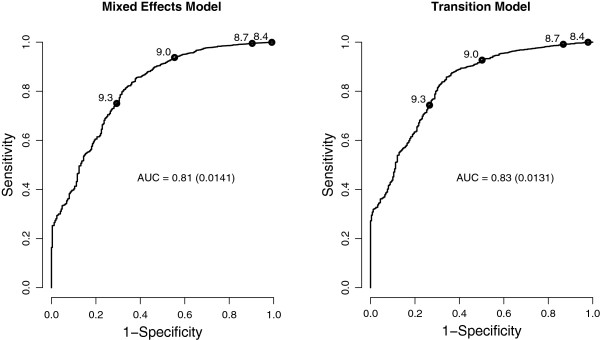
**ROC curves for male donors.** ROC curves of the prediction of eligibility for donation in male donors, for two different models. The standard errors of the AUCs are shown in parentheses. Different cut-off points for the predicted value are displayed on the curves.

Based on the fitted models, we calculated the predicted Hb levels for donors from the validation data set and predicted the eligibility (*Hb*>8.4 for men and *Hb*>7.8 for women) of a donor at a particular visit. Figure 3 displays the ROC curves for the 5th order transition model and the mixed effects model for male donors; since the results for female donors are similar, the ROC curves for female donors are not shown. All observations in the validation data set (n = 7,916 donors) were used to compute these ROC curves. The AUCs for the transition model and mixed effects model are 0.83 and 0.81 for men, respectively; for women we obtained AUC values of 0.73 and 0.72, respectively. The difference in AUCs between the two models is statistically significant (*p*-value <0.001), namely the transition model has a larger AUC than the mixed effects model and thus offers a better trade-off between sensitivity and specificity.

## Discussion

In this article, we presented transition models with different numbers of autoregressive terms and mixed effects models (a mixed effects model with random intercept for male donors and a mixed effects model with random intercept and random slope based on age for female donors), as plausible models to account for the dependence among subsequent Hb levels within a donor and as models to predict the future hemoglobin level. Based on the results for the validation data set, we showed that the transition model and the mixed effects model have almost the same predictive accuracy at the first few visits of a donor; however, for longer time series the transition model offers somewhat better predictions. To give an idea of the predictive performance, we have computed the ROC curve. Our results confirm that the transition model shows a small but significant improvement in the AUC compared to the mixed effects model.

Both the transition and the mixed effects models use the data of a person’s previous observations for making predictions. In the transition model only the last *q* observations are used for prediction the current response. However, in the mixed effects model, the empirical Bayes method for estimating a persons random effects uses all previous observations. Therefore, the mixed effects model requires more historical information than the transition model. Since the transition model is convenient in practice and needs less historical information compared to the mixed effects model, blood banks may use this model to predict the future hemoglobin level of a candidate and to determine which candidates should not be invited for the next donation.

Our approach of using transition or autoregressive models is quite novel in biomedical research, however in other fields such as econometrics, autoregressive modeling is a very well-known technique for tackling correlated financial phenomena and time series problems [27].

We do not claim that our final model is optimal; further research is needed to arrive at a better prediction model. First, the data set used in this paper is unbalanced in the sense that the time intervals between visits vary considerably, though this was not taken into account here. Second, there are more factors that are possibly associated with Hb level than those which we have investigated in this study, such as physical activity [28], race [29], nutrition [30] and smoking status [11,31]. Finally, the ultimate purpose of the prediction exercise is not the prediction of the future Hb value, but rather to determine the best time for the donor to return for donation. Hence, prediction models for Hb levels after blood donation should focus on the optimal timing of future donations, instead of on predicting future Hb levels. We are currently investigating such models.

## Conclusion

In this study we compared transition models and mixed effects models for predicting the Hb level in whole blood donors. The results showed that the transition model provides a somewhat better prediction than the mixed effects model, especially at high visit numbers. We believe that our paper shows the capabilities of using longitudinal models for prediction and that our findings may help reduce the number of deferred candidate in the blood banks.

## Competing interests

The authors declare that they have no competing interests.

## Authors’ contributions

KN analysed the data, and drafted the manuscript. EL, WdK, and JvR supervised and coordinated the project. MB extracted and processed the data, and AK revised the manuscript. All authors read and approved the final manuscript.

## Pre-publication history

The pre-publication history for this paper can be accessed here:

http://www.biomedcentral.com/1471-2288/13/62/prepub
